# In-Situ Study on Tensile Deformation and Fracture Mechanisms of Metastable β Titanium Alloy with Equiaxed Microstructure

**DOI:** 10.3390/ma15041325

**Published:** 2022-02-11

**Authors:** Jing Wang, Yongqing Zhao, Qinyang Zhao, Chao Lei, Wei Zhou, Weidong Zeng

**Affiliations:** 1 School of Materials Science and Engineering, Northwestern Polytechnical University, Xi’an 710072, China; wangjing127@mail.nwpu.edu.cn (J.W.); zengwd@nwpu.edu.cn (W.Z.); 2 Northwest Institute for Nonferrous Metal Research, Xi’an 710016, China; zhouwei2002563@163.com; 3 School of Materials Science and Engineering, Chang’an University, Xi’an 710064, China; zqy_ustb@163.com; 4 School of Materials Science and Engineering, Xi’an University of Technology, Xi’an 710048, China; leichao@xaut.edu.cn

**Keywords:** metastable β titanium alloy, equiaxed microstructure, dislocation slip, crack propagation, in-situ tensile test

## Abstract

Understanding the mechanisms of deformation and fracture of metastable β titanium alloys is of great significance for improving formability and service life. By combining the in-situ tensile test, TEM characterization and EBSD analysis, the tensile deformation behavior, activation of slip systems, crack initiation, and propagation of a high strength metastable β titanium alloy (Ti-5Cr-4Al-4Zr-3Mo-2W-0.8Fe) with equiaxed microstructure are investigated. The equiaxed microstructure is composed of primary α (α_p_) phase, transformed β (β_t_) matrix phase, and secondary α (α_s_) phase. In contrast to the hexagonal α_p_ grain with limited slip systems, the body-centered β_t_ matrix has more slip systems, however the hindering effect of α_s_ phases on dislocation slip leads to the different deformability of the α_p_ phase and β_t_ matrix. The equiaxed α_p_ grains are more prone to deformation and rotation to coordinate the overall deformation. The shear band leads to the formation of sub-grain boundary and even the fragmentation of α_p_ grains. As a result, the microvoids tend to nucleate at the grain boundary, phase interface, slip band, and shear band. The inhomogeneous deformation in the plastic deformation zone around the crack tip is the primary cause of damage. The crack propagation caused by microvoids coalescence advances along the grain boundaries and phase interfaces in the form of intergranular, and along the activated slip systems and shear bands in the form of transgranular. Pinpointing the situation in the equiaxed microstructure and combining that in other typical microstructures will help to summarize the universal deformation and fracture mechanisms of metastable β titanium alloy, and provide a basis for alloy design and microstructure tailoring.

## 1. Introduction

Titanium alloys are the fundamental materials in the fields of aerospace, marine, and medicine, and have been the long-standing research focus of material scientists [1]. In particular, metastable β titanium alloys have been widely applied in the structural components of the aviation industry due to their high specific strengths, better crack resistance, and excellent corrosion resistance [2,3]. Through a series of heat treatment and hot working, the microstructure of metastable β titanium alloys can be tailored to achieve the matching of high strength and well plasticity [4,5]. Compared with several typical microstructures, it is found that the equiaxed microstructure can help the alloys to achieve the above objective to the greatest extent [6]. Therefore, better understanding of the dependence of mechanical property and deformation capacity on the equiaxed microstructure is the key to improve the formability and service life of the alloys.

Recently, many works have been conducted on the deformation and fracture mechanisms of metastable β titanium alloys. Wang et al. [7,8] studied the tensile deformation and fracture behaviors of a new metastable β titanium alloy with single β phase and lamellar microstructure, successively. Firstly, the general mechanisms of deformation and fracture of metastable β titanium alloys was revealed by investigating the slip transfer and crack propagation at β grain boundaries; subsequently, the research scope was expended to slip transfer and crack propagation among various obstacles in the lamellar microstructure, including α lamella, β interlayer, phase interface, and grain boundary. Chen et al. [9] studied the effects of microstructure variables on the deformation and fracture mechanisms of the Ti-7333 alloy with bimodal microstructure, and found that the precipitation of secondary α phase significantly increases the density of the α/β phase interface and effectively hinders the dislocation movement, and the sole microvoids coalescence fracture mode and the mixed fracture mode respectively show relatively straight and tortuous crack propagation paths. These studies confirm that the microstructure and deformation degree determine the dominant deformation modes, including dislocation slip, stress-induced martensitic transformation, mechanical twinning, grain rotation and sliding, etc., and show the change of crack propagation modes when the crack meets different microstructures during the propagation process. However, the equiaxial microstructure is not covered. Liu et al. [10] ex-situ investigated the plastic deformation mode and slip transfer between the phase interface of the Ti-5Al-2.5Cr-0.5Fe-4.5Mo-1Sn-2Zr-3Zn alloy with equiaxed α grains, and revealed the dependence of deformation on the activation of slip systems with various Schmid factors (SF). In this work, the crystallographic orientation of the alloy was systematically characterized. However, further study on the relationship between crack propagation and crystallographic orientation could not be conducted unfortunately by ex-situ technology.

In-situ characterization techniques can observe the deformation, damage, and fracture of materials in real time, so they are widely used in titanium alloy research. Zhang et al. [11] observed the tensile deformation of the near-β titanium alloy Ti-17 with bimodal microstructure under in-situ scanning electron microscopy (SEM), and explored that the basal and prismatic slips are the dominant slip mode of α phase. Hémery et al. [12] studied the slip transfer in the Ti-6Al-4V alloy with a bimodal microstructure, and suggested that it is related to the grain boundaries and geometric compatibility factor m’. Jia et al. [13] researched the deformation mechanism of the Ti60 alloy with bimodal microstructure, and found that the prismatic slip system is the most easily activated in the equiaxed α grains, while the reason for the poor deformation ability of large lamellar α colonies is that there are relatively few slip systems. In addition to the in-situ SEM, the in-situ X-ray diffraction (XRD) [14], in-situ electron backscatter diffraction (EBSD) [15], in-situ transmission electron microscopy (TEM) [16], and other in-situ characterization techniques have also been used to study the real-time changes of phase composition, grain orientation, and dislocation configuration of titanium alloys during thermal-mechanical processing. In view of the excellent comprehensive properties of metastable β titanium alloys with equiaxed microstructure, it is of practical significance to in-situ study the phenomenon and mechanism of deformation and fracture in the equiaxed microstructure.

In this work, the tensile deformation and fracture mechanisms of a strength and plasticity well-matched metastable β titanium alloy Ti-5Cr-4Al-4Zr-3Mo-2W-0.8Fe (Ti-54432) with equiaxed microstructure are studied by an in-situ tensile test under SEM. Combined with TEM characterization and EBSD analysis, the microstructure changes during deformation, damage, and fracture were studied, including the activation of slip systems, the elongation, sliding and rotation of grains, the formation of shear band and sub-grain, the initiation and propagation of crack, as well as the morphology and composition of the fracture. Meanwhile, the underlying mechanism and influence rule of the deformation and fracture were discussed.

## 2. Experimental Procedure and Analysis Method

### 2.1. Material Preparation

**Figure 1:**
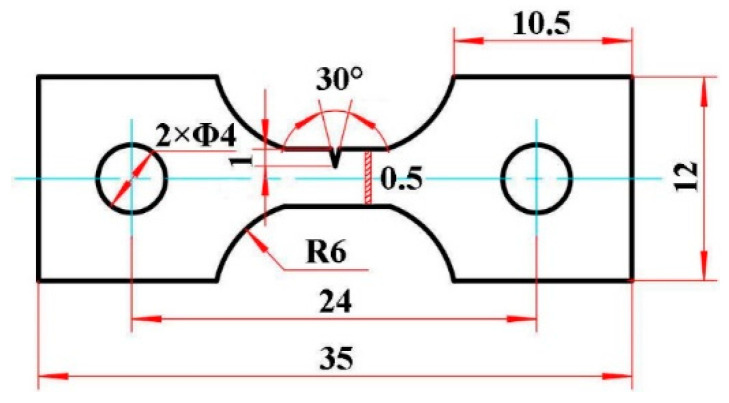
Sketch of shape and dimension of in-situ tensile test specimen (unit: mm).

The test material is a new high strength metastable β titanium alloy Ti-54432, which is produced by Northwest Institute for Nonferrous Metal Research, China. The chemical composition (wt.%) of this alloy is 4.10 Al, 5.33 Cr, 3.99 Zr, 2.63 Mo, 2.09 W, 0.83 Fe, 0.08 O, and balance Ti. A cast ingot was fabricated by the vacuum self-consuming arc-melting three times. The ingot was successively forged at 1150 °C and 950 °C (in β phase region) to refine the grains. Then the β-forged billet was re-forged at 830 °C (in a+β phase region). In each of the above three forging steps, the height reduction of the billet was more than 50%. The β transus temperature (T_β_) of the alloy was determined to be 860 ± 5 °C by the metallographic method. Some flakes were cut by wire EDM from the forged billet to prepare the tensile specimen, and the slow-feeding method of wire walking was adopted in the cutting process to ensure the surface quality of the specimen. Figure 1 shows the shape and dimension of in-situ tensile test specimen. To obtain the desired equiaxed microstructure, the tensile specimens were solution treated at 830 °C for 1 h plus air cooled to room temperature, and then aged at 600 °C for 6 h.

### 2.2. In-Situ Tensile Test and Microstructure Characterization

The in-situ tensile tests were conducted at a tensile speed of 0.05 mm/min in a Gatan loading auxiliary system installed on a Quanta FEG-450 SEM (FEI, Hillsboro, OR, USA). Several interruptions were performed during the in-situ tensile process to observe the microstructure evolution of the alloy, at this moment the tensile speed was reduced to 0. The observation area was framed near the tip of the V-notch on the specimen. The XRD analysis of the phase component of the alloy was conducted by a Bruker D8 Advance X-ray diffractometer (Bruker, Billerica, MA, USA) with Cu-Kα. The fracture morphology was observed by a field emission JSM-6460 SEM (JEOL, Showa, Tokyo, Japan) at an accelerating voltage of 15 kV. The crystallographic orientation was acquired by EBSD analysis combined with the HKL Channel 5 software (Oxford Instruments, Witney, Oxon, UK) at the acceleration voltage of 20 kV and a step size of 0.7 μm. The EBSD specimens were electrochemically polished in an electrolyte mix of perchloric acid and glacial acetic acid with a volume proportion of 1:16 at a voltage of 60 V, an electric current of 0.7 A, and a dwelling time of 30 s. The microstructure characteristics were characterized by a JEM-200 CX TEM (JEOL, Showa, Tokyo, Japan) at 200 kV, and the TEM specimens were taken from the severe plastic deformation zone near the fracture on the tensile specimen. In other words, the farther away from the fracture, the less plastic deformation, thus the sampling location should be as close to the fracture as possible. In detail, some flakes were cut by wire EDM, and sanded down to about 40 μm. Then some 3-mm diameter disks were cut from the flakes and twin-jet electropolished in a solution of 60% methanol, 35% butanol, and 5% perchloric acid (in volume) at 30 V and −20 °C.

### 2.3. Identification of Activated Slip System

The activated slip systems were identified by analyzing the slip trace and the calculated value of the corresponding SF [17]. Since the dislocation is difficult to move in <*c*+*a*> type slip systems with large critical shear stress, only the activation of <*a*> type slip systems in the α phase is considered here. The crystallographic orientation of α grain in the interest region was determined by EBSD data analysis. The SF is used to evaluate the difficulty of slip system’s activation, and the calculation formula of its value is [11,18,19]:

(1) $$\mathrm{SF}=\frac{nT_{c}}{\left|n\right|\cdot \left|T_{c}\right|}\cdot \frac{sT_{c}}{\left|s\right|\cdot \left|T_{c}\right|},$$

where, *n* represents the unit vector in the normal direction of slip plane, *s* represents the unit vector in the slip direction, and *T_c_* represents the unit vector in the stress axis direction in crystal coordinates. The larger the SF value of a slip system is, the easier it is to be activated, and the slip system with the largest SF value is the principal slip system.

In addition, there is an effect of the angle between the slip plane in a specific slip system and the stress axis on the activation of the slip systems. Therefore, whether the slip system is activated, can be judged by comparing the calculated value *θ_c_* with the measured value *θ_m_* (from SEM image) of this angle when analyzing the slip traces, and the calculation formula of *θ_c_*:

(2) $$\theta_{c}=\arccos\frac{S_{T}T_{c}}{\left|S_{T}\right|\cdot \left|T_{c}\right|},$$

where, *S_T_* is the unit vector of the intersecting line between the slip plane and the specimen observation surface. *S_T_* = *n* × *N_c_*, where *N_c_* is the unit vector in the normal direction of the specimen observation surface. In general, the criterion of ±5° is used to match *θ_c_* and *θ_m_*. If the deviation between *θ_c_* and *θ_m_* is less than 5°, the slip system is determined to be activated, while if the deviation is greater than 5°, the slip system is considered to be inhibited.

## 3. Results

### 3.1. Tensile Deformation

#### 3.1.1. Stress-Strain Relationship

**Figure 2:**
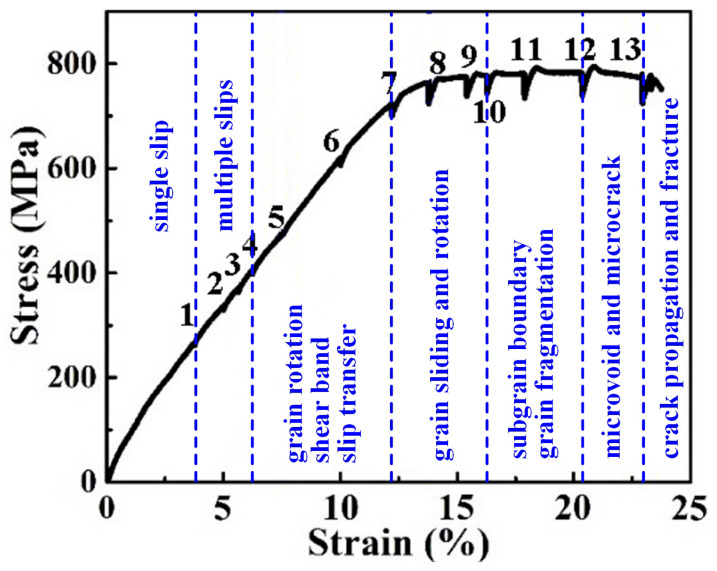
Stress-strain curve of the Ti-54432 alloy in the in-situ tensile test with several interruptions.

Figure 2 illustrates the stress-strain relationship of the Ti-54432 alloy during the in-situ tensile test with several interruptions. The curves can be divided into elastic deformation segment, plastic deformation segment, and transient fracture segment. In the plastic deformation stage, due to the selection of the specimen with notch and the stress release during the interruptions of the in-situ tensile test, the stress value on the tensile curve is relatively low. In fact, the yield strength, tensile strength, and elongation of the Ti-54432 alloy with equiaxed microstructure measured by a uniaxial tensile test are about 1122 MPa, 1148 MPa, and 20%, respectively. The reason for the smaller stress of a notched specimen in the in-situ tensile test than that of a standard specimen in the uniaxial tensile test is that the internal stress states of the two specimens under uniaxial tension are not same. In general, the shear strength of metal materials is much lower than the tensile strength. The stress state near the notch is no longer a simple uniaxial tensile stress state, the existence of shear stress leads to more deformation of the specimen. Even so, it also can be seen from the stress-strain relationship during tensile deformation that the strength and plasticity of the Ti-54432 alloy with equiaxed microstructure are well matched.

#### 3.1.2. Deformation and Rotation of Grains

**Figure 3:**
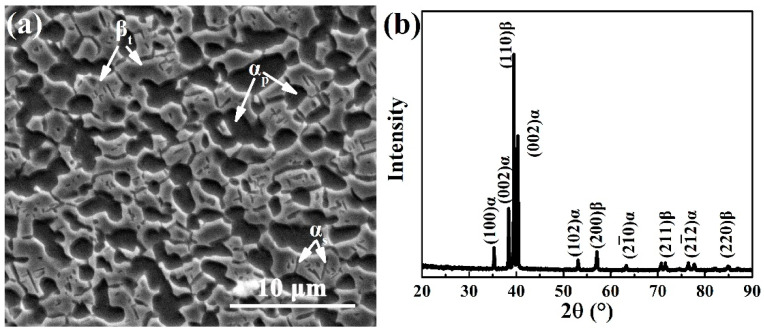
SEM image of microstructures (**a**) and XRD pattern (**b**) of the Ti-54432 alloy after solution and aging treatments.

Figure 3 shows the initial microstructure of the Ti-54432 alloy before the in-situ tensile test. In the SEM image, not only the equiaxed primary α (α_p_) phase and the transformed β (β_t_) matrix phase, but also a small quantity of the short rod-like secondary α (α_s_) phase on the β_t_ matrix can be observed. The XRD pattern confirms the coexistence of α and β phases, and the quantitative analysis shows that the α phases account for 55.4% (volume fraction) and the remaining 44.6% is accounted by the β phases. Due to the low solution temperature, a large number of α_p_ grains are formed during the solution process, which inhibit the nucleation and precipitation of the α_s_ phase on β_t_ matrix during the subsequent aging process, thus the amount of the α_s_ phase is small.

**Figure 4:**
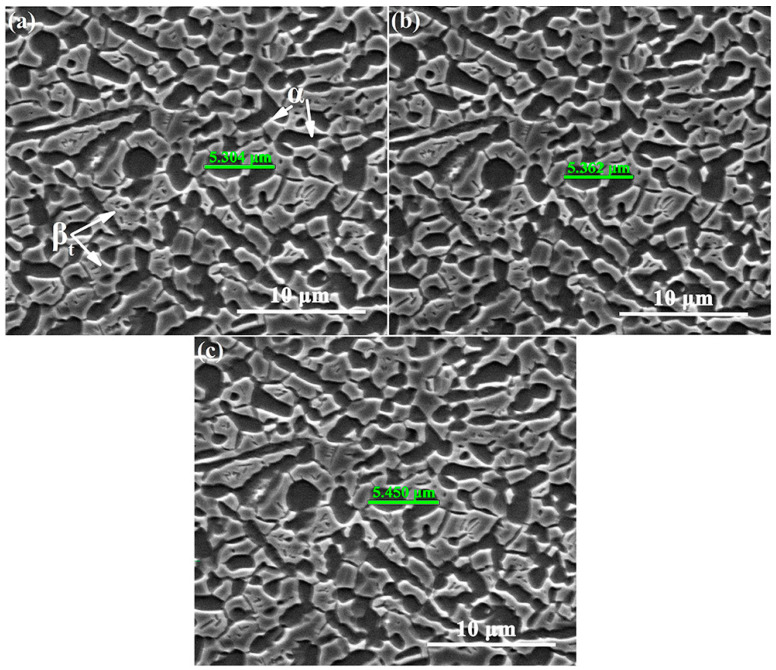
SEM images of microstructures of the Ti-54432 alloy with different strains in the in-situ tensile test: (**a**) 4.8%; (**b**) 5.9%; and (**c**) 7.5%.

Figure 4 shows the microstructure evolution of the Ti-54432 alloy during the in-situ tensile test, from which the size change of the equiaxed α_p_ grains can be measured. Two adjacent equiaxed α_p_ grains are labeled as the measurement objects, and the length between the farthest two points in the two grains are measured. The initial distance between the two points in the tensile specimen with a strain of 4.8% is 5.304 μm. When the strain increases to 5.9% and 7.5%, the distance between the two points becomes 5.362 μm and 5.450 μm, respectively. In other words, the strains of equiaxed grains are respectively about 1.09% and 2.75% when the corresponding strain increments of the specimen are 1.1% and 2.7%. The strain of equiaxed α_p_ grains and the macroscopic strain of alloy are very close, indicating that the deformation of the alloy is mainly contributed by the deformation of equiaxed α_p_ grains, and the adjacent grains elongate along the tensile direction simultaneously to coordinate the deformation [9].

**Figure 5:**
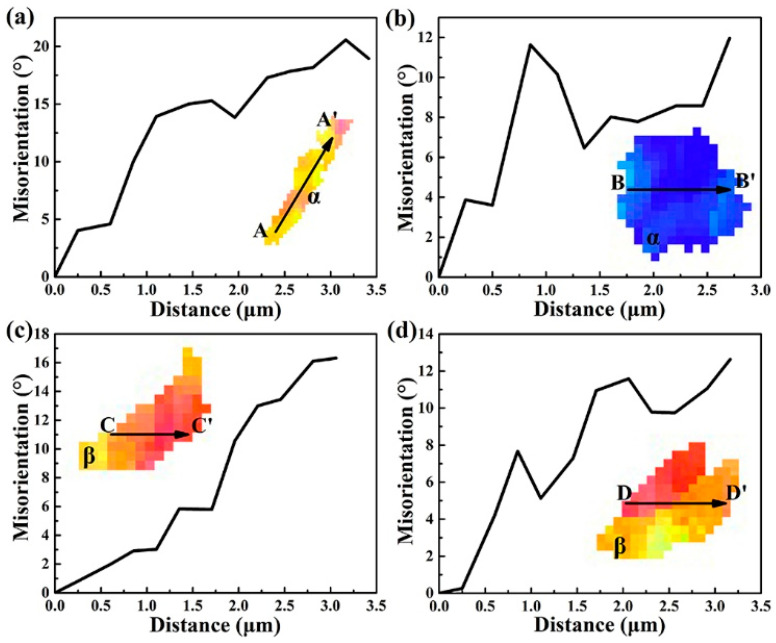
Misorientation of selected four grains in the Ti-54432 alloy after the in-situ tensile test: (**a**) α grain 1; (**b**) α grain 2; (**c**) β grain 1; and (**d**) β grain 2.

In the process of tensile deformation, the substructure evolution in the grain depends on the cumulative (maximum) misorientation [20,21,22]. The orientation inside the grain is consistent before deformation. Four grains in the deformed Ti-54432 alloy are selected for crystallographic orientation analysis, including two α grains and two β grains, as shown in Figure 5. For the two α grains, their cumulative misorientations, namely the misorientations between two pixels along the arrow direction (A-A’ and B-B’), are 20° and 12°, respectively. This indicates that the α grains rotate along the arrow direction and seriously deform by the dislocation slip, accumulating abundant dislocations within the grains. For the two β grains, the cumulative misorientations (C-C’ and D-D’) are 18° and 12°, respectively, indicating that the β grains also rotate. As a result, the large angular rotation and serious deformation adjust the grains to a crystallographic orientation that is more conducive to the dislocation slip, but also provide the possibility for the nucleation of microvoids at the grain boundary.

### 3.2. Damage and Fracture

#### 3.2.1. Damage Evolution

**Figure 6:**
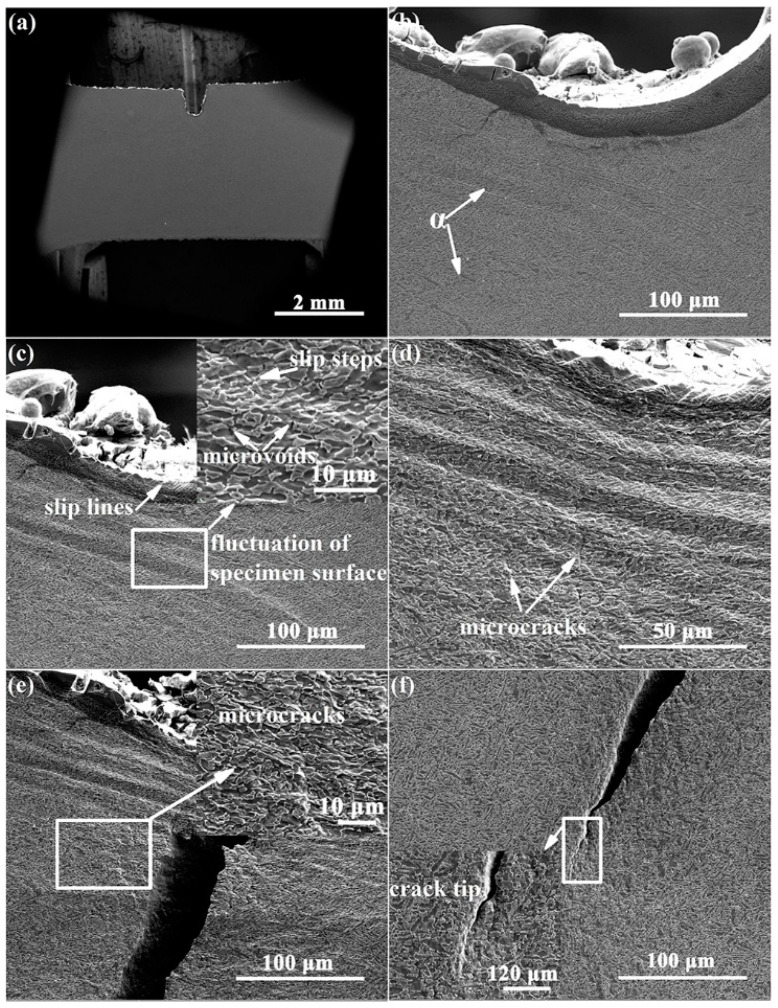
SEM images of deformation and damage of the Ti-54432 alloy with different strains in the in-situ tensile test: (**a**) 0; (**b**) 2.9%; (**c**) 5.0%; (**d**) 7.5%; (**e**) 10.1%; and (**f**) 11.7%.

Figure 6 presents the damage evolution of the Ti-54432 alloy during the in-situ tensile test. When obvious plastic deformation occurs, some slip lines appear near the tip of the V-notch, and the surface of the specimen changes from flat to rough, showing the characteristic of fluctuation. The sliding and rotation of α and β grains result in the nucleation of microvoids at the phase interfaces. As the deformation continues, the stress concentration and inhomogeneous deformation near the V-notch intensify, and the microvoids grow up and gradually coalesce into microcracks. Several microcracks in the shear band near the tip of the V-notch converge to form a principal crack, which then expands rapidly to split the specimen. Although the overall crack propagation path is relatively straight, the local magnification of the crack tip region shows that the crack actually has several slight deflections during propagation. At the same time, more grains around the V-notch participate in deformation, and the severe plastic deformation in the crack initiation region makes the specimen surface more uneven. Some secondary microcracks appear in the deformation zone on both sides of the principal crack.

#### 3.2.2. Fracture Mode

**Figure 7:**
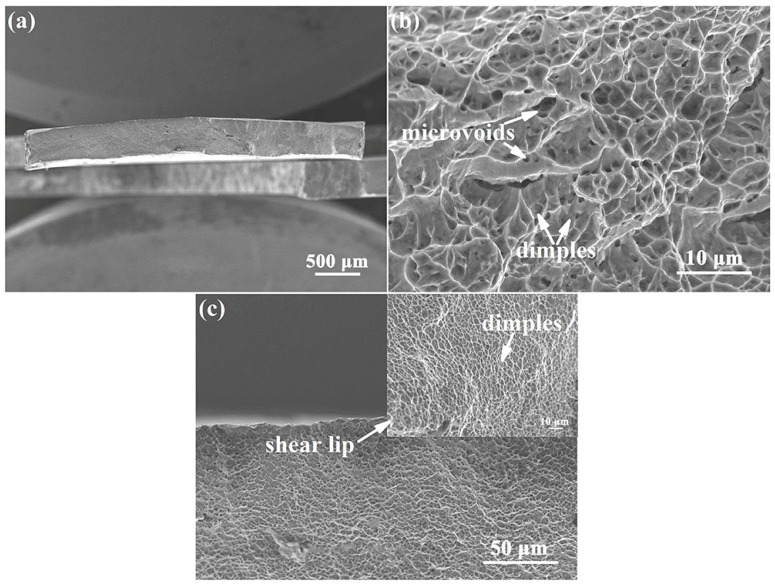
SEM images of fracture morphology of the Ti-54432 alloy after the in-situ tensile test: (**a**) overall appearance; (**b**) microvoids and dimples; and (**c**) shear region.

Figure 7 displays the fracture morphology of the Ti-54432 alloy after the in-situ tensile test. The fracture surface is relatively flat and consists of many regular dimples. Many microvoids can be found at the bottom and edges of the dimples, and the microcracks are formed by the coalescence of several adjacent microvoids. In the region where shearing occurs, the shear band consists of some small and shallow dimples at an angle of 45° to the tensile direction. These features suggest that the Ti-54432 alloy with equiaxed microstructure exhibits excellent ductility, and its fracture mode is the microvoids coalescence fracture.

## 4. Discussion

### 4.1. Deformation Mechanism

#### 4.1.1. Activation of Slip Systems

Table 1 lists seven activated slip systems in α_p_ grains identified by the SF calculation and slip trace analysis, and the corresponding grains are labeled in the inset figure. These activated slip systems can be classified to two pyramidal slip systems, two basal slip systems and three prismatic slip systems. The activation of slip systems inside grain depends on the relationship between the crystallographic orientation of grain and the loading direction; for example, the slip system with the normal direction perpendicular to the loading direction is easily activated [23]. In the process of tensile deformation, obvious slip lines appear in both α and β phases. The basal slip systems and prismatic slip systems are proved to be more easily activated due to their relatively small critical resolved shear stresses (CRSS) and the high SF values [14,17,24]. In contrast, the <*a*> pyramidal slip systems with high CRSS are usually difficult to activate unless there is a large angle rotation of the α_p_ grain in which the pyramidal slip is located [25]. However, the reality is that some slip systems are still inactivated due to the inappropriate crystallographic orientation of the α_p_ grains even when these grains undergo severe plastic deformation. That is, the crystallographic orientation of the equiaxed α_p_ grains and its relationship to the stress axis determine whether and which internal slip systems can be activated.

#### 4.1.2. Deformation Characteristics

**Figure 8:**
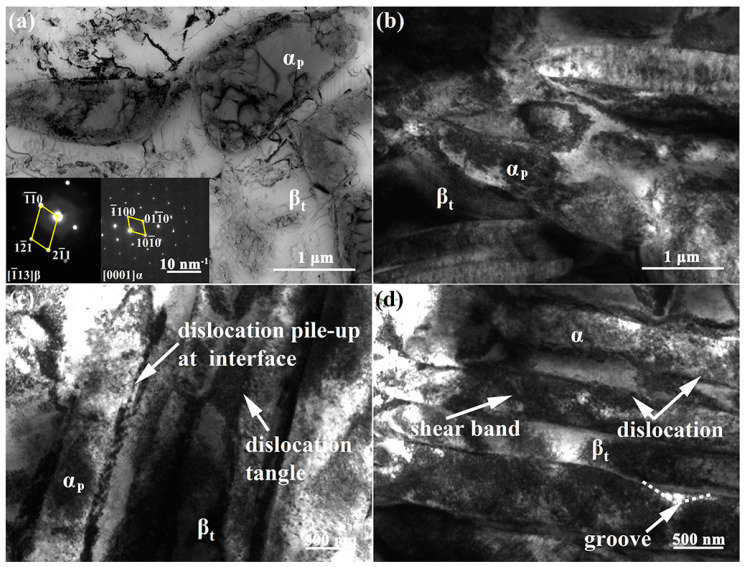
TEM images of microstructure deformation characteristics of the Ti-54432 alloy in the in-situ tensile test: (**a**) initial microstructure; (**b**) elongated α grains and β_t_ matrix; (**c**) dislocation pile-up and tangle; and (**d**) shear band in the α grain.

Figure 8 reveals some deformation characteristics in the microstructure of the Ti-54432 alloy in the in-situ tensile test. It can be seen that there are fewer dislocations in the equiaxed α_p_ grains and β_t_ matrix of undeformed alloy, and the diffraction patterns prove the existence of α and β phases. After tensile deformation, the equiaxed α_p_ grains are obviously elongated in the tensile direction, and there are many dislocations at the grain boundaries. Although the β_t_ matrix has more slip systems, the hindering effect of α_s_ phases on dislocation slip leads to the formation of many dislocation tangles in the β_t_ matrix. When the slip transfer between the α_p_ grains and β_t_ matrix is insufficient, the shear bands are generated in the severely deformed α_p_ grain, resulting in the fragmentation of α_p_ grains into smaller grains [26,27]. It can be inferred that although there are more potential slip systems in the β phase than in the α phase, the α_p_ grains are relatively softer and more prone to the dislocation slip because the precipitation of the α_s_ phase in the β_t_ matrix increases the strength of the β_t_ matrix and inhibits the dislocation slip [10,28]. The above reasoning can also be confirmed by the fact that the deformation of α accounts for most of the total deformation of the alloy. However, for the two-phase titanium alloy TC21, the β matrix is still relatively soft and deformed first, even if the α_s_ phase precipitates on the β matrix [29]. We believe that the hardness contrast and deformability difference between α and β phases may depend on the degree of precipitation strengthening, that is, the size, quantity and distribution of the α_s_ phase.

**Figure 9:**
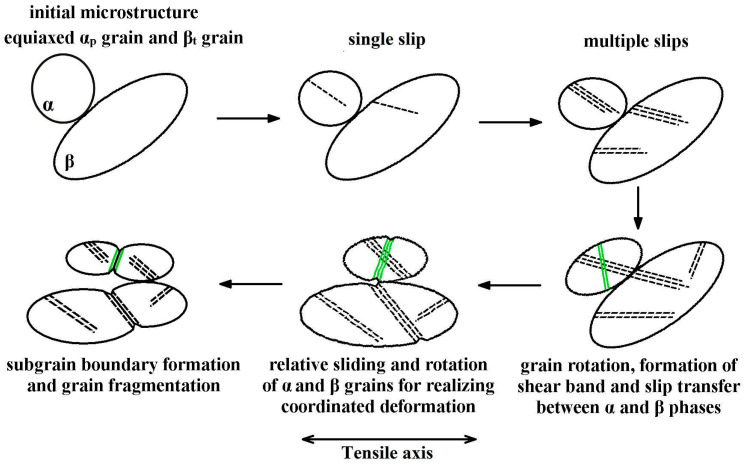
Schematic diagram of grains deformation and slip transfer in the Ti-54432 alloy during the tensile process. The meanings of different patterns: grain of the black circle, slip line of the black dotted line, shear band of the green line, and subgrain boundary of the black line. Omitting the α_s_ phase.

To intuitively understand the morphologic evolution of grains and the slip transfer at the grain boundary in the Ti-54432 alloy during tensile deformation, a schematic diagram is drawn as shown in Figure 9. At the beginning of tensile deformation, single slip occurs in the equiaxed α_p_ grains, and the crystallographic orientation of the grains can satisfy the deformation driven by a slight dislocation movement. Subsequently, further deformation leads to the increase of slip lines and the activation of multiple slip systems, and the deformation incompatibility between α and β phases forces the α_p_ grains to rotate [30]. At this time, the dislocation moving to the grain boundary and the change of grain orientation realize the slip transfer from the α phase to β phase, so that the β grains also undergo a dislocation slip-induced deformation [31,32]. Meanwhile, because the β phase has a relatively large number of slip systems [33], some internal dislocations can pass through the phase interface, realizing the slip transfer from the β phase to α phase, so that the deformation incompatibility between two phases can be reduced. When the multiple slips cannot meet the requirement of further deformation, the shear band is formed in the severely deformed α_p_ grain. To coordinate the overall deformation, both α and β grains rotate and slide relative to each other, making that the microvoids are easy to nucleate at the slip band, shear band, and grain boundary [34]. As the deformation continues, the slip band and shear band extend, forming subgrain boundaries and finally splitting grains into several small grains.

### 4.2. Fracture Mechanism

#### 4.2.1. Cause of Damage

**Figure 10:**
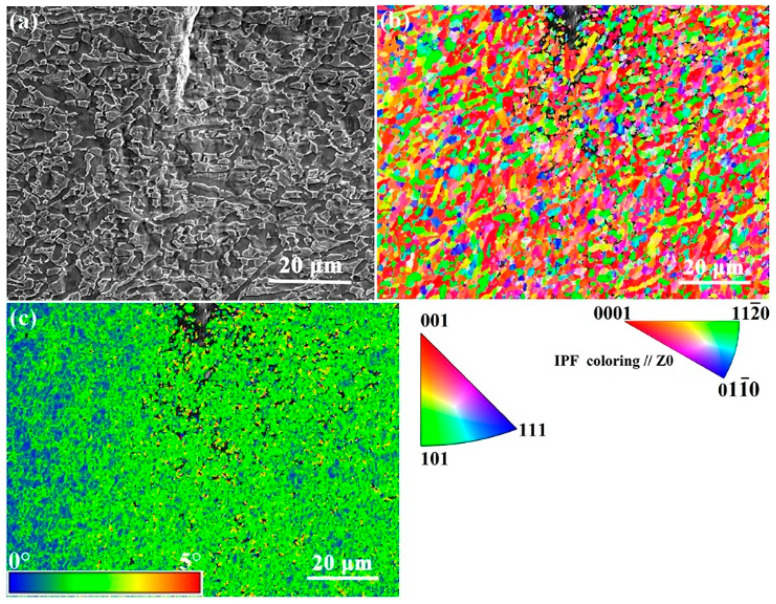
SEM images of crack tip in the Ti-54432 alloy with strain of 13.4% in the in-situ tensile test: (**a**) morphology; (**b**) inverse pole figure (IPF) map; and (**c**) Kernel average misorientation (KAM) map.

Figure 10 displays the microstructure of the plastic deformation zone around the crack tip of the Ti-54432 alloy in the in-situ tensile test. The crystallographic orientations of the α_p_ grains and β_t_ matrix are analyzed by EBSD, and the deformation features of the crack tip plastic zone is revealed in the inverse pole figure (IPF) map and Kernel average misorientation (KAM) map. The corresponding KAM values near the crack tip are high, especially at the α_p_/α_p_ grain boundary, α_p_/β_t_ phase interface, and α_s_/β_t_ phase interface, which indicate that there is obvious inhomogeneous deformation in this region [35]. In contrast, the deformation of the region far from the crack tip is relatively small, and the corresponding KAM values are low. The stress state in the plastic deformation region around the crack tip is different from that in other regions, namely that it is no longer simply a state of uniaxial tensile stress, so severe inhomogeneous deformation and stress concentration phenomena will occur, leading to the formation of microvoids and microcracks more easily ahead of the crack tip.

#### 4.2.2. Fracture Characteristics

**Figure 11:**
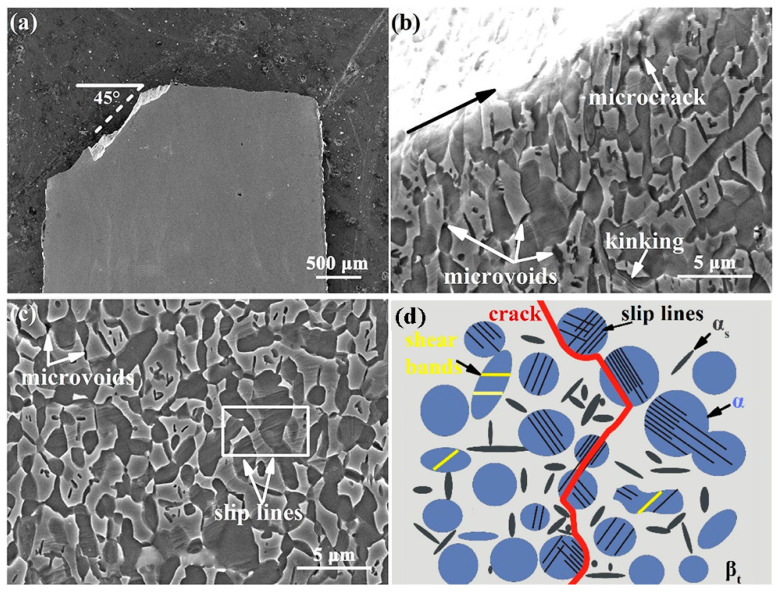
SEM images of crack propagation of the Ti-54432 alloy in the in-situ tensile test: macroscopic characteristics of the overall path (**a**), microscopic characteristics of the local path (**b**), microstructure around the path (**c**), and schematic diagram of crack propagation (**d**). The meanings of different patterns in the schematic diagram are: equiaxed α_p_ grains of the blue circle, β_t_ matrix of the gray background, slip lines of the black line, shear bands of the yellow line, α_s_ phases of short black rods, and crack propagation path of the red zigzag line.

The morphology, crystallographic orientation, and internal activated slip systems of the α and β phases in the equiaxed microstructure affect the crack propagation behavior, and thus determine the fracture mode of the alloy, as shown in the SEM images in Figure 11. A schematic diagram is summarized to visually show the responses of crack to different microstructures during propagation. The crack propagates from the V-notch to the middle of the specimen along the direction at an angle of 45° with the tensile direction, namely the maximum shear stress direction, and then along the direction almost perpendicular to the tensile direction until the specimen fracture. The deflection of the crack propagation direction is determined by the crystallographic orientation of grains along the propagation path and the activated slip systems within the grains [7]. Dislocation accumulation at grain boundaries of equiaxed α_p_ grains promotes the activation of slip systems in the adjacent β_t_ matrix [36]. When there are activated slip systems in both α_p_ and β_t_ phases, the slip transfer may occur between them. We propose that the slip transfer is an important mode of the coordinated deformation of two phases, since the continuous slip lines are observed in the equiaxed α_p_ grains and adjacent β_t_ matrix [37]. At the same time, because the potential path of slip is extended, it may also become the path for transgranular crack propagation. In this case, the crack does not deflect obviously and propagates rapidly when it passes through the equiaxed α_p_ grain and β_t_ matrix. However, even though multiple slip systems are activated in the β_t_ matrix, it is difficult for slip transfer to occur between two adjacent grains if the crystallography orientations of the α_p_ and β grains are severely mismatched. In the propagation process, the crack will turn to the grain with the more suitable crystallographic orientation and activated slip systems. Predictably, once the well matching relationships of the crystallography orientation between the grains in the crack propagation direction are restored, the crack will continue to straight propagate rapidly. Besides the transgranular propagation in the grains, the crack also propagates in the intergranular mode at the interfaces with a large number of microvoids and microcracks, including the α_p_/α_p_ grain boundary, α_p_/β_t_ phase interface, and α_s_/β_t_ phase interface. For the same microvoids coalescence fracture, the crack propagation may be only transgranular in the bimodal microstructure [9], while it is usually a mixture of transgranular and intergranular in the equiaxed microstructure.

### 4.3. Limitations and Implications

#### 4.3.1. Limitations of the Work

At present, there are two limitations that need to be further considered. Firstly, the in-situ study of deformation and fracture is incomplete. In-situ tensile tests are observed under SEM and can monitor some phenomena related to fracture, such as damage generation and development, crack initiation and propagation, and fracture morphology. However, the microstructure responses related to deformation during tensile process, such as dislocation multiplication and slip, mechanical twinning, shear band, and sub-grain formation, are lacking in real time, which may require in-situ TEM characterization. Secondly, the dependence of deformation and fracture on the stress state is unclear. In recent years, a large number of studies have shown that the mechanical properties of titanium alloys under tensile and compressive stresses are obviously different. However, the current in-situ tests are carried out under tensile stress, and the deformation and fracture behaviors of alloys under compressive stress are not well understood. Supplementing the in-situ studies under compressive stress may be able to better explain the tension and compression asymmetry of titanium alloys during deformation.

#### 4.3.2. Implications for Future Research

In future, there are two implications that may be reflected in the following work. Firstly, the universal mechanisms of deformation and fracture of metastable β titanium alloys should be summarized and refined. Together with previous works, the deformation and fracture mechanisms under several typical microstructures have been revealed. Among them, some deformation modes and influencing factors are shared, such as the dislocation slip and crystallographic orientation, which may be the decisive factors affecting the mechanical properties of the alloys. For these, a targeted in-depth study may be closer to the physical nature behind the phenomenon. Secondly, the quantitative study on the relationship between the microstructure characteristic parameters and the deformation and fracture related performance indexes needs to be conducted. Just as there is a controversy about which α phase or β phase is more difficult to deform, we hypothesize that the key problem lies in the volume fraction of the α_s_ phases precipitated on the β matrix. Due to the difference in the number of potential slip systems, the pure β phase is bound to deform more easily than the α phase. With the increase of the volume fraction of α_s_ phases, the precipitation strengthening effect on the β matrix is enhanced, and the strength of the β phase increases, approaching or even surpassing the strength of the α phase. In addition to the volume fraction, both the equiaxed α grains and α lamellae, their size is also a key factor affecting the mechanical properties of alloys. Therefore, clarifying the quantitative relationship is the premise of microstructure tailoring aimed at properties optimization.

## 5. Conclusions

The tensile deformation and fracture mechanisms of a high strength metastable β titanium alloy (Ti-54432) with equiaxed microstructure were investigated by in-situ tensile tests. The microstructure responses during deformation, damage, and fracture were characterized and analyzed, including the activation of slip systems, the deformation, sliding and rotation of grains, the formation of shear band and sub-grain, the initiation and propagation of crack, as well as the morphology and composition of the fracture. The main findings can be drawn as follows:

1. The initial microstructure of the alloy before deformation is mainly composed of equiaxed α_p_ grains and β_t_ matrix, and there is the short rod-like α_s_ phase precipitated in the β_t_ matrix. Although the body-centered β phase has more potential slip systems than the hexagonal α phase, the precipitation strengthening of the α_s_ phase makes the β_t_ matrix more difficult to deform. Therefore, the deformation of α_p_ grains in the main contribution to the overall deformation of the alloy, is mainly characterized by elongation, rotation, and fragmentation.
2. The basal slip systems and prismatic slip systems are the main activated slip systems in equiaxed α_p_ grains. At the beginning of deformation, the original grain orientation and single slip can satisfy the deformation requirements. As the deformation increases, the multiple slips are activated and the grains rotate and slide. The adjusted crystallography orientation is helpful to the slip transfer between the two phases and the coordination of the overall deformation. Under large deformation, the shear bands are formed in α grains, and slip bands are formed in β grains, which develop into sub-grain boundaries.
3. Under severe deformation, the stress concentration and inhomogeneous deformation in the plastic deformation zone around the crack tip are the primary cause of damage. The microvoids nucleate at the grain boundary, phase interface, slip band, and shear band, and then grow and coalesce into microcracks, which then expand into a primary crack. The crack propagates along the activated slip systems and shear bands in the form of transgranular, and also along the grain boundaries and phase interfaces in the form of intergranular. The conversion of the two forms depends on the orientation relationship of the grains along the propagation path and the activation of slip systems within the grains.

## Figures and Tables

**Table 1:** Activated slip systems in α_p_ grains.

No.	Euler Angle (°)	SF	*θ*_c_ (°)	*θ*_m_ (°)	Activated Slip System	
1#	(82.3, 109.4, 6.1)	0.18	45	44	$\left(\bar{1}101\right)\left[11\bar{2}0\right]$	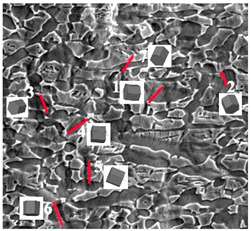
2#	(24.8, 57.0, 42.6)	0.48	105	108	$\left(\bar{1}011\right)\left[1\bar{2}10\right]$	
3#	(106.1, 140.0,16.6)	0.48	108	104	$\left(0001\right)\left[1\bar{2}10\right]$	
4#	(37.1, 91.8, 48.9)	0.46	40	37	$\left(0001\right)\left[\bar{2}110\right]$	
5#	(160.4, 71.6, 14.9)	0.49	90	90	$\left(1\bar{1}00\right)\left[11\bar{2}0\right]$	
6#	(179.4, 99.6, 23.6)	0.48	100	97	$\left(\bar{1}100\right)\left[11\bar{2}0\right]$	
7#	(154.9, 131.4, 0.2)	0.44	60	65	$\left(10\bar{1}0\right)\left[1\bar{2}10\right]$	

## Data Availability

Some or all data and images generated or used during the present work are available from the corresponding authors by reasonable request.

## References

[1] Banerjee D., Williams J.C. (2013). Perspectives on Titanium Science and Technology. Acta Mater..

[2] Gao J., Huang Y., Guan D., Knowles A.J., Ma L., Dye D., Rainforth W.M. (2018). Deformation mechanisms in a metastable beta titanium twinning induced plasticity alloy with high yield strength and high strain hardening rate. Acta Mater..

[3] Dong R., Li J., Kou H., Fan J., Tang B. (2019). Dependence of mechanical properties on the microstructure characteristics of a near β titanium alloy Ti-7333. J. Mater. Sci. Tech..

[4] Li C., Chen J., Li W., Ren Y.J., He J.J., Song Z.X. (2016). Effect of heat treatment variations on the microstructure evolution and mechanical properties in a b metastable Ti alloy. J. Alloy. Compd..

[5] Fan J., Zhang Z., Gao P., Yang R., Li H., Tang B., Kou H., Zhang Y., Esling C., Li J. (2020). On the nature of a peculiar initial yield behavior in metastable β titanium alloy Ti-5Al-5Mo-5V-3Cr-0.5Fe with different initial microstructures. J. Mater. Sci. Tech..

[6] Tanii S., Umezawa O., Yamabe-Mitarai Y. (2021). Equiaxed α microstructure evolution in wrought Ti-10Al-1Zr-1Mo-1Nb alloy during annealing. J. Alloy. Compd..

[7] Wang J., Zhao Y., Zhou W., Zhao Q., Huang S., Zeng W. (2021). In-situ investigation on tensile deformation and fracture behaviors of a new metastable β titanium alloy. Mater. Sci. Eng. A.

[8] Wang J., Zhao Y., Zhou W., Zhao Q., Lei C., Zeng W. (2021). In-situ study on tensile deformation and damage evolution of metastable β titanium alloy with lamellar microstructure. Mater. Sci. Eng. A.

[9] Chen N., Kou H., Wu Z., Qiang F., Hua K., Fan J., Tang B., Li J., Molina-Aldareguia J.M. (2021). Microstructural sensitivity and deformation micro-mechanisms of a bimodal metastable β titanium Ti–7Mo–3Nb–3Cr–3Al alloy. Mater. Sci. Eng. A.

[10] Liu X., Qian Y., Fan Q., Zhou Y., Zhu X., Wang D. (2020). Plastic deformation mode and α/β slip transfer of Ti–5Al-2.5Cr-0.5Fe-4.5Mo–1Sn–2Zr–3Zn titanium alloy at room temperature. J. Alloy. Compd..

[11] Zhang S., Zeng W., Zhao Q., Ge L., Zhang M. (2017). In situ SEM study of tensile deformation of a near-β titanium alloy. Mater. Sci. Eng. A.

[12] Hémery S., Nizou P., Villechaise P. (2018). In situ SEM investigation of slip transfer in Ti-6Al-4V: Effect of applied stress. Mater. Sci. Eng. A.

[13] Jia R., Zeng W., Zhao Z., Zhang P., Xu J., Wang Q. (2022). In-situ investigation on the deformation mechanism of duplex microstructure of a near α titanium alloy. J. Alloy. Compd..

[14] Zhang D., Wang L., Zhang H., Maldar A., Zhu G., Chen W., Park J.S., Wang J., Zeng X. (2020). Effect of heat treatment on the tensile behavior of selective laser melted Ti-6Al-4V by in situ X-ray characterization. Acta Mater..

[15] Lilensten L., Danard Y., Brozek C., Mantri S., Castany P., Gloriant T., Vermaut P., Sun F., Banerjee R., Prima F. (2019). On the heterogeneous nature of deformation in a strain-transformable beta metastable Ti-V-Cr-Al alloy. Acta Mater..

[16] Yao T., Du K., Wang H., Huang Z., Li C., Li L., Hao Y., Yang R., Ye H. (2017). In situ scanning and transmission electron microscopy investigation on plastic deformation in a metastable β titanium alloy. Acta Mater..

[17] Bridier F., Villechaise P., Mendez J. (2005). Analysis of the different slip systems activated by tension in a α/β titanium alloy in relation with local crystallographic orientation. Acta Mater..

[18] Huang S., Zhao Q., Lin C., Wu C., Zhao Y., Jia W., Mao C. (2021). In-situ investigation of tensile behaviors of Ti–6Al alloy with extra low interstitial. Mater. Sci. Eng. A.

[19] Tan C., Sun Q., Xiao L., Zhao Y., Sun J. (2018). Characterization of deformation in primary α phase and crack initiation and propagation of TC21 alloy using in-situ SEM experiments. Mater. Sci. Eng. A.

[20] Gao X., Zeng W., Wang Y., Long Y., Zhang S., Wang Q. (2017). Evolution of equiaxed alpha phase during heat treatment in a near alpha titanium alloy. J. Alloy. Compd..

[21] Izadi E., Darbal A., Sarkar R., Rajagopalan J. (2017). Grain rotations in ultrafine-grained aluminum films studied using in situ TEM straining with automated crystal orientation mapping. Mater. Des..

[22] Zhang Z., Lunt D., Abdolvand H., Wilkinson A.J., Preuss M., Dunne F.P.E. (2018). Quantitative investigation of micro slip and localization in polycrystalline materials under uniaxial tension. Int. J. Plast..

[23] Zhang C., Li H., Eisenlohr P., Liu W., Boehlert C.J., Crimp M.A., Bieler T.R. (2015). Effect of realistic 3D microstructure in crystal plasticity finite element analysis of polycrystalline Ti-5Al-2.5Sn. Int. J. Plast..

[24] Li H., Mason D.E., Bieler T.R., Boehlert C.J., Crimp M.A. (2013). Methodology for estimating the critical resolved shear stress ratios of α-phase Ti using EBSD-based trace analysis. Acta Mater..

[25] Yan Z., Wang K., Zhou Y., Zhu X., Xin R., Liu Q. (2018). Crystallographic orientation dependent crack nucleation during the compression of a widmannstätten-structure α/β titanium alloy. Scr. Mater..

[26] Hua K., Wan Q., Zhang Y., Kou H., Zhang F., Li J. (2021). Crystallography and microstructure of the deformation bands formed in a metastable β titanium alloy during isothermal compression. Mater. Charact..

[27] Zhang C.J., Guo C.X., Zhang S.Z., Feng H., Chen C.Y., Zhang H.Z., Cao P. (2020). Microstructural manipulation and improved mechanical properties of a near α titanium alloy. Mater. Sci. Eng. A.

[28] Shi R., Nie Z., Fan Q., Li G. (2016). Elastic plastic deformation of TC6 titanium alloy analyzed by in-situ synchrotron based X-ray diffraction and microstructure based finite element modeling. J. Alloy. Compd..

[29] Lei L., Zhao Q., Zhao Y., Huang S., Wu C., Jia W., Zeng W. (2021). Study on the intrinsic factors determining impact toughness of TC21 alloy. Mater. Charact..

[30] Jha J.S., Toppo S.P., Singh R., Tewari A., Mishra S.K. (2021). Deformation behavior of Ti-6Al-4V microstructures under uniaxial loading: Equiaxed Vs. transformed-β microstructures. Mater. Charact..

[31] Zhou Y., Wang K., Yan Z., Xin R., Wei S., Wang X., Liu Q. (2020). Ex-situ study on mechanical properties and deformation mechanism of three typical microstructures in TA19 titanium alloy. Mater. Charact..

[32] Bhattacharyya J.J., Nair S., Pagan D.C., Tari V., Lebensohn R.A., Rollett A.D., Agnew S.R. (2021). Elastoplastic transition in a metastable β-Titanium alloy, Timetal-18—An in-situ synchrotron X-ray diffraction study. Int. J. Plast..

[33] Barriobero-Vila P., Gussone J., Kelm K., Haubrich J., Stark A., Schell N., Requena G. (2018). An in situ investigation of the deformation mechanisms in a β-quenched Ti-5Al-5V-5Mo-3Cr alloy. Mater. Sci. Eng. A.

[34] Jiang Y.-Q., Lin Y.C., Jiang X.-Y., He D.-G., Zhang X.-Y., Kotkunde N. (2020). Hot tensile properties, microstructure evolution and fracture mechanisms of Ti-6Al-4V alloy with initial coarse equiaxed phases. Mater. Charact..

[35] Huang S., Zhao Q., Zhao Y., Lin C., Wu C., Jia W., Mao C., Ji V. (2021). Toughening effects of Mo and Nb addition on impact toughness and crack resistance of titanium alloys. J. Mater. Sci. Tech..

[36] Joseph S., Bantounas I., Lindley T.C., Dye D. (2018). Slip transfer and deformation structures resulting from the low cycle fatigue of near-alpha titanium alloy Ti-6242Si. Int. J. Plast..

[37] Li H., Cai W. (2018). Understanding the deformation mechanism of individual phases of a dual-phase beta type titanium alloy using in situ diffraction method. Mater. Sci. Eng. A.

